# Structural elucidation of a polysaccharide from *Flammulina velutipes* and its immunomodulation activities on mouse B lymphocytes

**DOI:** 10.1038/s41598-018-21375-0

**Published:** 2018-02-15

**Authors:** Wen-Han Wang, Jing-Song Zhang, Ting Feng, Jing Deng, Chi-Chung Lin, Hua Fan, Wen-Juan Yu, Hai-Ying Bao, Wei Jia

**Affiliations:** 1 National Engineering Research Center of Edible Fungi, Key Laboratory of Applied Mycological Resources and Utilization of Ministry of Agriculture, Shanghai Key Laboratory of Agricultural Genetics and Breeding. Institute of Edible Fungi, Shanghai Academy of Agricultural Sciences, Shanghai, 201403 China; 2 College of Chinese Traditional Medicine, Jilin Agricultural University, Jilin, Changchun, 130118 China; 3 Key Laboratory of Culinary Science, Sichuan Tourism University, Chengdu, 610100 China; 4 Institut für Laboratoriumsmedizin Klinische Chemie und Pathobiochemie; Campus Virchow Klinikum; Charite-Universitätsmedizin, Berlin, 13353 Germany; 5 Instrumental Analysis Center, Shanghai JiaoTong University, Shanghai, 200240 China

## Abstract

A novel polysaccharide FVPB2 was purified from fruiting bodies of *Flammulina velutipes*. Its structure was elucidated by monosaccharide composition and methylation analyses, UV-Visible and FTIR spectroscopy as well as NMR. FVPB2 was a homogeneous heteropolysaccharide (molecular weight ~ 1.50 × 10^4^ Da) containing D-galactose, D-mannose, L-fucose, and D-glucose at molar ratio of 1.9:1.2:1:2.5. *In vitro* immunomodulatory studies showed FVPB2 induced proliferation of mouse spleen lymphocytes in a dose-dependent manner. The levels of IgM and IgG, secreted by B cells, increased after FVPB2 treatment. So FVPB2 has potential to be a new important immunomodulatory nutraceutical.

## Introduction

For thousands of years, mushrooms have been known as an important source of nutritional diet and medicine. Extensive studies have revealed that many species of mushrooms have promising potential in improving human health and preventing diseases^1^. The search for active components and investigation of the functional mechanisms of natural products used in fungal medicine are becoming increasingly important. Polysaccharides, as one of the most important components isolated from mushrooms, have been correlated with multiple pharmacological activities, such as antioxidant, immunomodulatory, and reducing blood lipid^2^. Hitherto, about 200 active polysaccharides have been isolated from mushrooms^3–5^. All these activities are due to enhancement of the immune function of the human body.

*Flammulina velutipes* (Curtis) Singer (*F*. *velutipes*), owing to its high nutritional values and attractive taste is one of the most popular edible fungi in China and Japan. Its production and consumption ranks the fourth in the edible fungi^6,7^. Therefore, most studies have been focused on the conventional nutrient profiles of *F*. *velutipes*, such as amino acids, vitamins and bioactive macromolecules, and increasing production to obtain the maximum benefit. The first study of *F*. *velutipes* polysaccharide (FVP) was reported by Kamasuka *et al*.^8^. Since then several other polysaccharides have been isolated from *F*. *Velutipes* fruiting bodies^9,10^. Many reports have indicated that the bioactivities of polysaccharides can be affected by various factors including structure, molecular weight, monosaccharide compositions, even the extraction and isolation methods^11,12^. For example, a polysaccharide isolated from *F*. *velutipes* was proven to exert anti-inflammatory effect by decreasing CD4^+^, CD8^+^, ICAM-1, and MPO levels in serum and on burn injury in male Wistar rats^13^. *F*. *velutipes* mycorrhizae polysaccharide was isolated and activated immune function of mice T cell lymphocyte^14^. Results showed that the proportions of CD3^+^ and CD4^+^ T lymphocytes; the ratio of CD4^+^/CD8^+^ and the levels of interlukin-2 (IL-2) and tumor necrosis factor-α (TNF-α) were significantly increased after being fed with polysaccharides from *F*. *velutipes* mycorrhizae.

Recently, many studies have revealed that *F*. *velutipes* polysaccharides (FVP) can stimulate the immune system^1–3,15^. However, almost all the reports verified that *F*. *velutipes* polysaccharides have effect on T cells. Based on this background, we attempt to test whether active polysaccharides from FVP have effect on B cell lymphocytes. The objective of this study was to elucidate the chemical structure of the novel *F*. *velutipes* polysaccharide FVPB2 and investigate its bioactivities on mouse B lymphocytes.

## Results

### Composition and structural characterization of FVPB2

The high performance size exclusion chromatography (HPSEC) profile showed that FVPB2 eluted as a single symmetrical peak, indication it was a homogeneous polysaccharide. Its molecular weight was determined to be ~1.50 × 10^4^ Da based on the calibration graph prepared with pullulan standards. Lack of absorption at 280 nm and 260 nm in the ultraviolet-visible (UV) scanning spectrum indicated that FVPB2 did not contain protein and nucleic acid. Sugar analysis and GC-MS revealed the presence of galactose (Gal), mannose (Man), fucose (Fuc), and glucose (Glc) at a molar ratio of 1.9:1.2:1:2.5.

The FVPB2 exhibited absorption bands at 3416, 2931, 1651, and 1405 cm^−1^ on the Fourier Transform Infrared (FT-IR) spectrum. The broadly stretched intense peak at 3416 cm^−1^ was due to the hydroxyl stretching vibration of the polysaccharide. The band at 2931 cm^−1^ was due to C-H stretching vibration. The relatively strong absorption peak at 1651 cm^−1^ and the weak one at 1405 cm^−1^ also indicated the characteristic IR absorption of polysaccharides. No absorption peaks at 1730 cm^−1^ indicated that there were no uronic acids^16^.

Methylation analysis of polysaccharides was performed to determine the substitution pattern of monosaccharide residues. The fully methylated products were hydrolyzed, converted into partially methylated alditol acetates (PMAAs), and analyzed by Gas Chromatography-Mass Spectrometry (GC-MS). On the basis of the GC-MS data, the polysaccharide was found to contain a branched structure with mannopyranose residue and fucopyranose residue at the terminals as well as with 3,4-di-substituted galactose, 1,2-di-substituted galactose, 1,6-di-substituted glucose, and 3,6-di-substituted glucose constituting the main chain, and a glucose residue at the other terminal.

**Figure 1:**
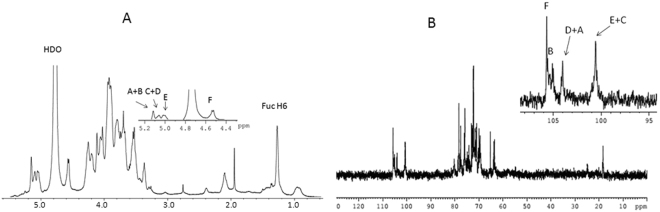
500 MHz ^1^H NMR and 125 MHz ^13^C NMR spectrum of FVPB2 polysaccharide in deuterium oxide (D_2_O) at 27 °C. The anomeric protons are labeled as (**A**–**F**). (**A)**. 500 MHz ^1^H NMR spectrum of FVPB2 polysaccharide in D_2_O at 27 °C. The anomeric protons are labeled as (**A**–**F**). (**B**) 125 MHz ^13^C NMR spectrum of FVPB2 polysaccharide in D_2_O at 27 °C).

The results of ^1^H nuclear magnetic resonance (NMR) at 500Mz (Fig. 1A), ^13^C NMR (125 MHz, Fig. 1B), and ^13^C-^1^H heteronuclear multiple quantum coherence (HMQC) analyses indicated that the repeating unit of the polysaccharide comprised six sugar residues, which were designated as A-F in the ^1^H NMR spectrum according to the decreasing chemical shifts of the anomeric protons.

The ^1^H NMR spectrum of FVPB2 (Fig. 1A) at 27 °C mainly contained signals for six anomeric protons at δ 4.58 to δ 5.17. One signal at 1.28 (*J*_H-5_, _H-6_ = 6.42 Hz) corresponded to the ^1^H signal for CH_3_-C group of Fuc^17^. Other signals of sugar protons were in the region of δ 4.49 to δ 3.25.

The peaks in the ^1^H NMR spectrum (Fig. 1A), which was supported by both ^13^C NMR (Fig. 1B) and ^1^H -^13^C HMQC showed six anomeric signals at δ 5.17 (single broad peak (br.s), δ 5.16 (br.s), δ 5.11 (br.s), δ 5.09 (br.s), 5.06 (br.s), and δ4.58 (double peaks (d), *J*_H-1, H-2_ = 10 Hz), were designated as A-F, corresponding to the signals at δ 104.02, 105.58, 100.50, 104.10, 100.59, and 105.69, respectively, in the ^13^C NMR spectrum. Furthermore, a signal at δ 18.36 upfield revealed a CH_3_-C group (C-6 of Fuc)^16^.

The identities of the monosaccharide residues A-F were established based on 2D-NMR spectra analysis involving ^1^H-^1^H correlated spectroscopy (COSY), total correlation spectroscopy (TOCSY), nuclear overhauser effect spectroscopy (NOESY), and ^1^H-^13^C HMQC, which were used to assign the chemical shifts and anomeric configurations of the six sugar residues present in the repeating unit.

Residue A has an anomeric signal at δ 5.17. The proton chemical shifts from H-1 to H-6 for residue A were assigned from COSY, TOCSY and HMQC spectra (Table 1). Identification of the mannopyranose residue A signals were in accordance with the small value of *J*_H-1, H-2_ < 3 Hz and the large coupling constant value of *J*_H-4, H-5_ (~8 Hz). The small *J*_H-1, H-2_ value for the D-mannosyl residue did not provide information about the anomeric configuration^18^. The α configuration of mannose residue was inferred from the value of *J*_C-1, H-1_ = 170 Hz in HMBC^19^. Except for the downfield shift of C-1 (δ 104.02), no carbon signal was evident within the δ 76–82 range indicating that A was a terminal α-D-mannopyranose^20^_._

**Table 1:** ^1^H and ^13^C NMR chemical shifts (ppm) of FVPB2 at 27 °C.

Residue	Proton or carbon					
	H-1/C-1	H-2/C-2	H-3/C-3	H-4/C-4	H-5/C-5	H-6/C-6
A α-D-Man*p*(1 →	5.17	4.12	3.91	4.06	3.82	3.56^a^, 3.70^b^
	104.02	72.73	71.48	69.48	76.19	65.21
B - → 3,4)- α-D-Gal*p*(1 →	5.16	3.98	**4**.**04**	**4**.**22**	3.91	3.80^a^, 3.96^b^
	105.69	73.07	**80**.**09**	**77**.**65**	71.52	63.76
C → 6)-α-D-Glc*p*(1 →	5.11	3.84	3.74	3.94	4.07	**3**.**95**^**a**^, **3**.**79**^b^
	100.50	76.07	73.07	72.11	72.51	**69**.**50**
D α-L-Fuc*p*(1 →	5.09	3.89	4.12	3.91	4.23	1.28
	104.10	71.52	72.73	71.52	69.91	18.36
E → 2)-α-D-Gal*p*-(1 →	5.06	**3**.**90**	4.07	3.93	4.27	3.68^a^, 3.94^b^
	100.59	**80**.**26**	72.23	72.15	71.52	65.24
F → 3,6)-β-D-Glc*p*-(1 →	4.58	3.38	**3**.**56**	3.69	3.91	**4**.**26**^**a**^, **3**.**92**^b^
	105.58	75.76	**78**.**30**	71.35	71.52	**71**.**52**

^a^Chemical shift for H-6a.

^b^Chemical shift for H-6b.

Using similar approaches, the spin system with the H-1 (B) signal at δ 5.16 was obtained from the complete proton and carbon chemical shifts (Table 1). The H-3 and H-4 signals of residue B overlapped due to similar chemical shifts. The downfield shifts of the C-3 (δ 80.09) and C-4 (δ 77.65) carbon signals with respect to the standard values for galactopyranose indicated that residue B was 1, 3, 4- α-D-Gal*p*.

The H-1 signal at high field (δ 5.11) and a small value of *J*_H-1, H-2_ = 0 indicated that residue C is an α-linked residue^21^. Proton chemical shifts from H-2 to H-6 were assigned from 2D NMR, including COSY, TOCSY, NOESY, HMBC and HMQC spectra. The large *J*_H-2, H-3_ value and *J*_H-3, H-4_ coupling constants (9 Hz) and the typical H-1, H-2, and H-4 intra-correlations in the NOESY spectrum pointed to residue C as D-glucopyranose^21^. The downfield chemical shift of the C-6 (δ 69.50) carbon signal with respect to standard values for glucopyranoses indicated that residue C is a 1,6-link α-D glucopyranose.

The anomeric signal at δ 5.09 and the small *J*_H-1, H-2_ value indicated that residue D is an α-linked residue. The ^1^H resonances for H-2, H-3, and H-4 of this residue were assigned from COSY, TOCSY, and NOESY spectra. H-5 and H-6 were assigned from the ^1^H-^1^H COSY spectrum. The cross-peak of H-6 and C-4 in the HMBC spectrum unambiguously showed that H-5 and H-6 were located in residue D. The proton chemical shift for the methyl group at δ 1.28 indicated an α-fucose residue. Both carbon and proton chemical shifts were typical of 6-deoxyhexopyranose, and residue D can only be L-fucose since this sugar was the only deoxyglucose identified by the GC-MS analysis. Except for the downfield shift of C-1 (δ 104.10), no carbon signal was evident within the δ 76–82 range indicating that residue D is a terminal alpha-L-Fuc*p*.

Residue E had an anomeric signal at δ 5.06 and a very small *J*_H-1, H-2_ value, indicating an α-linked residue. The cross-peak δ 5.06/3.90 was detected in the COSY spectrum and, since δ 5.06 corresponded to H-1, the δ 3.90 signal was assigned to H-2. The ^1^H resonances for H-3 were assigned from the cross-peaks in the COSY and TOCSY spectra. The H-4 and H-5 resonances were assigned from the H-3/H-4 and H-4/H-5 cross-peaks in the NOESY spectrum. The H-6a and H-6b resonances were obtained from the COSY spectrum. The large coupling constant value *J*_H-3, H-4_ (~8.0 Hz) and the small coupling constant value *J*_H-4, H-5_ (<3.0 Hz) suggested that residue E is a galactopyranose. The downfield shift of the C-2 (δ 80.26) carbon signal with respect to the standard value for galactopyranose indicated that residue E is (1,2)-α-D-Gal*p*.

The H-1 signal at high field (δ 4.58) and large *J*_H-1, H-2_ coupling constant indicated that residue F is a β-linked residue. Proton chemical shifts from H-2 to H-6 were assigned from 2D NMR, including COSY, TOCSY, NOESY, HMBC and HMQC spectra. The large *J*_H-2, H-3_ and *J*_H-3, H-4_ coupling constants (9 Hz) and the typical H-1, H-2, and H-4 intra-correlations in the NOESY spectrum pointed out that residue F was D-glucopyranose^22^. The downfield shifts of the C-3 (δ 78.30) and C-6 (δ 71.52) carbon signal with respect to standard values for glucopyranoses indicated that residue F was a 1,3,6-link β-D Glc*p*.

The sequence of the glycosyl residues was determined from NOESY studies followed by confirmation by HMBC experiments. Inter-residue NOESY correlations were observed between H-1 of residue A and H-3 of residue B, between H-1 of residue B and H-6 of residue C, between H-1 of residue D and H-6 of residue F, as well as between H-1 of residue F and H-2 of residue E. Between H-3 of residue B and H-1 of residue A, Between H-4 of residue B and H-1 of residue E, Between H-6 of residue C and H-1 of residue B, Between H-2 of residue E and H-1 of residue F, Between H-6 of residue F and H-1 of residue D. HMBC experiments demonstrated clear correlations between H-4 of residue B and C-1 of residue E, between H-2 of residue E and C-1 of residue F, between H-1 of residue F and C-2 of residue E, as well as between H-3 of residue F and C-1 of residue C.

**Figure 2:**
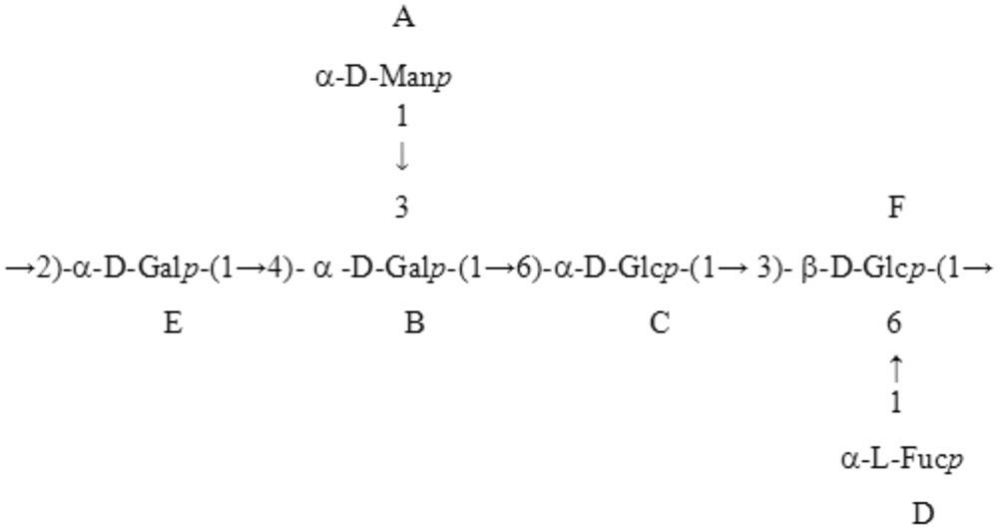
Repeating unit of FVPB2.

Based on the data presented above, polysaccharide FVPB2 has the following repeating unit (Fig. 2).

### Effect of FVPB2 on proliferation of mouse spleen lymphocytes

**Figure 3:**
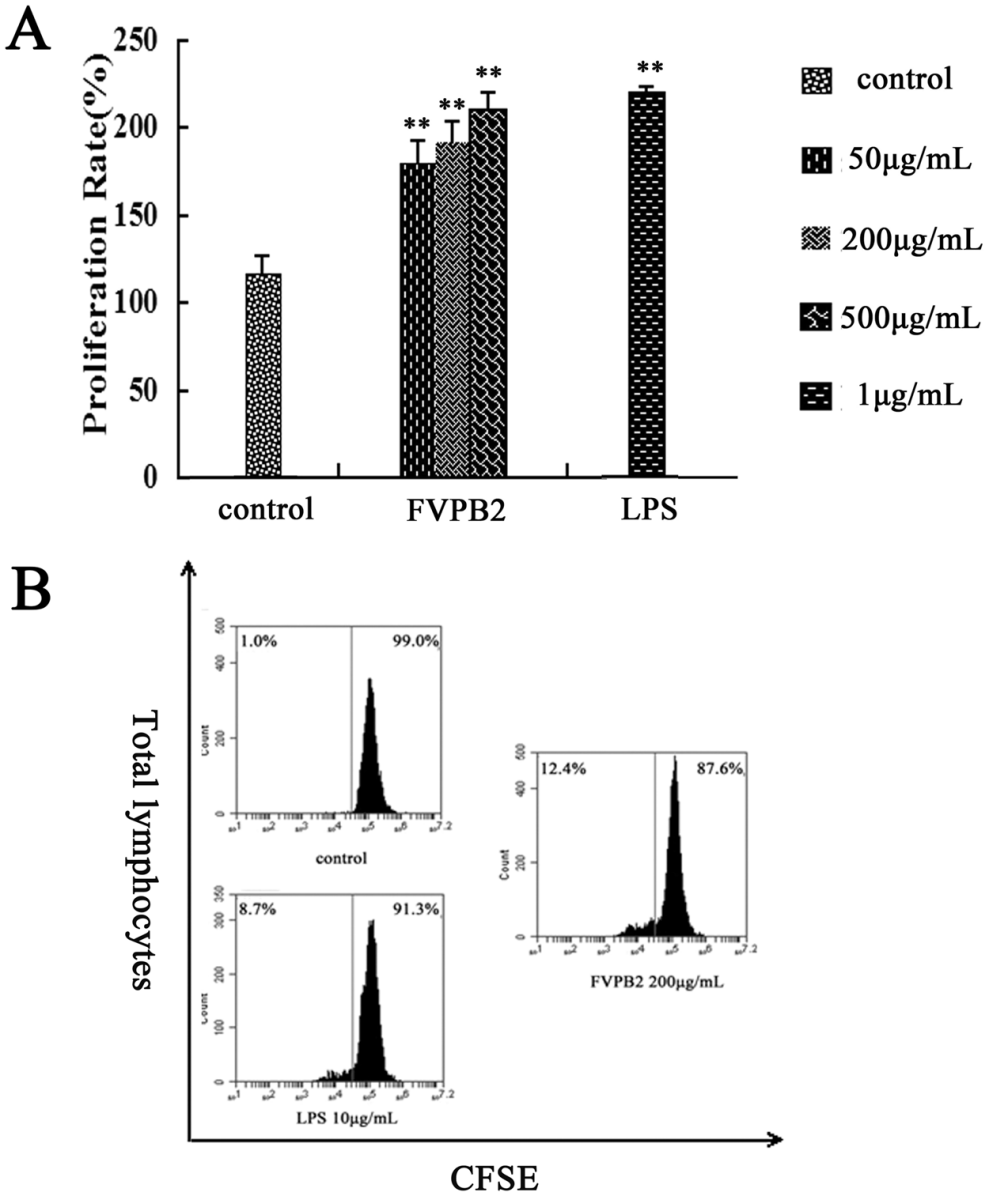
Proliferation of mouse spleen lymphocytes were stimulated by FVPB2. (**A**) Cells were treated by FVPB2 at the indicated concentration for 72 h (*P < 0.05 or **P < 0.01, vs. control); (**B**) After stimulation by 200 μg/mL FVPB2 for 48 hours, analysis of lymphocytes was performed by FACS. Phosphate-buffered saline (PBS) and medium containing 10 μg/mL LPS served as the negative and positive controls respectively.

Lymphocytes were prepared from the mouse spleens and treated with various concentrations of FVPB2. As shown in Fig. 3A, FVPB2 stimulated the proliferation of mouse spleen lymphocytes in a dose-dependent manner. The results also showed that proliferation rate of lymphocytes treated with 500 μg/mL FVPB2 was similar to that of lipolysaccharide (LPS) (1 μg/mL).

Carboxyfluorescein succinimidyl ester (CFSE) is an intracellular fluorescent dye that dilutes 2-fold when a cell divides^22^. Cells are typically labeled with CFSE *in vitro* and labeled cells can be followed there after *in vitro* or *in vivo*. As shown in Fig. 3B, the proliferation rates of the control and FVPB2 treated groups (200 μg/mL) were 1.03% ± 0.35% and 15.67% ± 3.00% respectively.

Our experimental results indicated that the proliferation of lymphocytes treated with 200 μg/mL FVPB2 increased by 14.64% as compared to control. Furthermore, the enhancement of cell proliferation by FVPB2 was better than that of B cells treated with LPS (13.9% vs. 4.64%).

### Identification of activation of B lymphocytes treated by FVPB2

**Figure 4:**
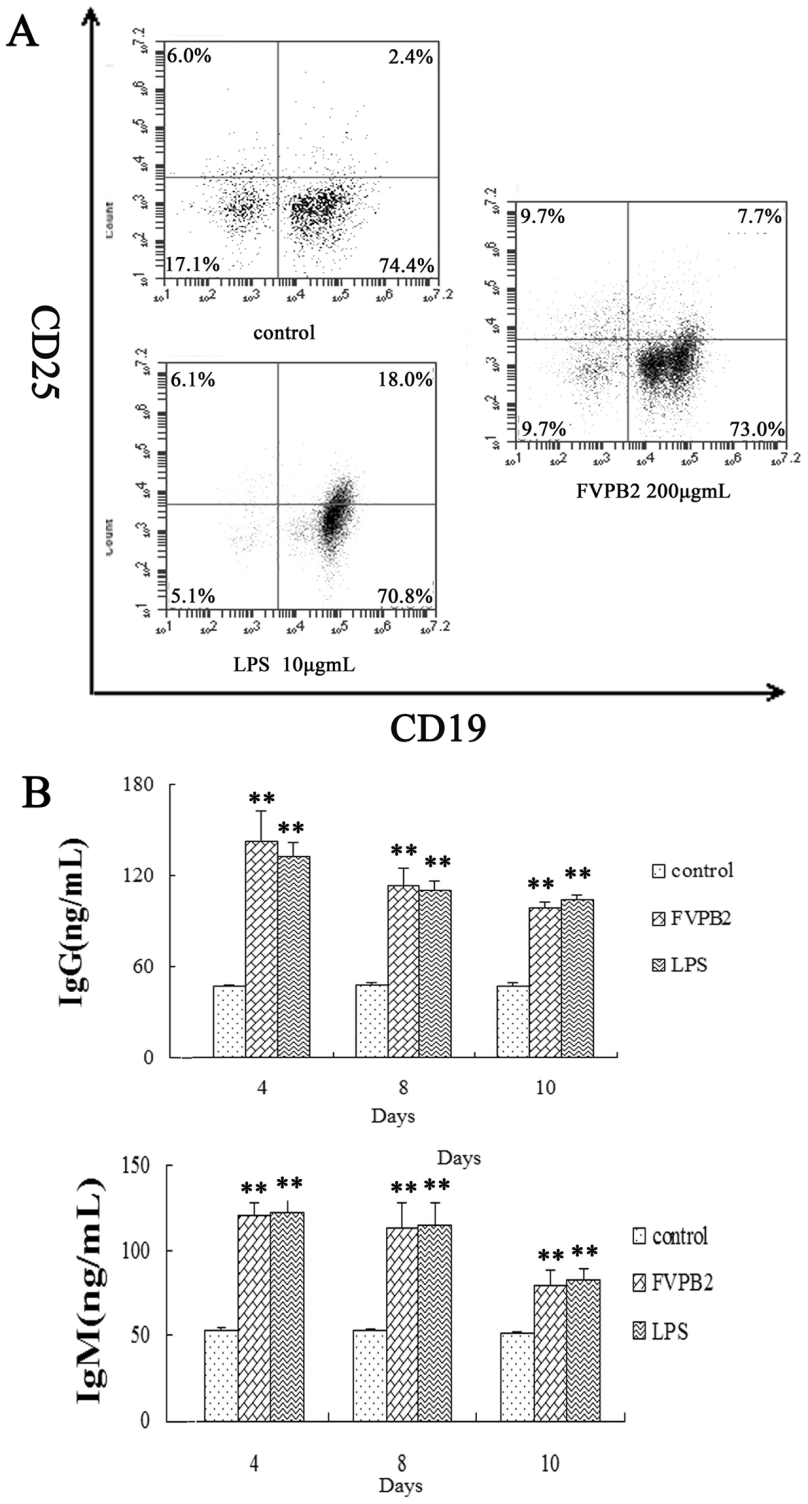
Identification of activation of B lymphocytes treated by FVPB2. (**A**) The activated B lymphocytes were identified with anti-CD19-PE and anti-CD25-APC. (**B**) Secretion of IgG and IgM by mouse spleen lymphocytes incubated with FVPB2. Mouse spleen lymphocytes were incubated with 200 μg/mL FVPB2, 10 μg/mL LPS or PBS. Each value represents the mean ± SD of separate triplicate experiments. Values were significantly higher than those of controls are indicated by *P <0.05 and **P <0.01).

After mouse spleen lymphocytes had been stimulated by FVPB2, the mouse spleen lymphocytes were double stained either with anti-CD19-PE and anti-CD25-APC to identify the stimulated sub-population of lymphocytes. Compared with the control, the activated B cells (CD19^+^/CD25^+^) ratio increased after treatment with FVPB2 (Fig. 4A). In addition, some of the lymphocytes treated by FVBP2 enlarged by electron microscopy.

Effects of FVPB2 on levels of Immunoglobulin G (IgG) and Immunoglobulin M (IgM) antibodies secreted from B cells were examined by enzyme-linked immunosorbent assay (ELISA). Compared with the control, IgG and IgM secretion increased in mouse spleen lymphocytes treated with 200 μg/mL FVPB2 at 4, 8 and 10 days (Fig. 4B). The results indicated that the release of IgG and IgM peaked at the forth days after treatment by FVPB2, and the levels of these two immunoglobulins decreased with longer treatment time.

### Effect of FVPB2 on the proliferation of B cells isolated from mouse spleen lymphocytes

**Figure 5:**
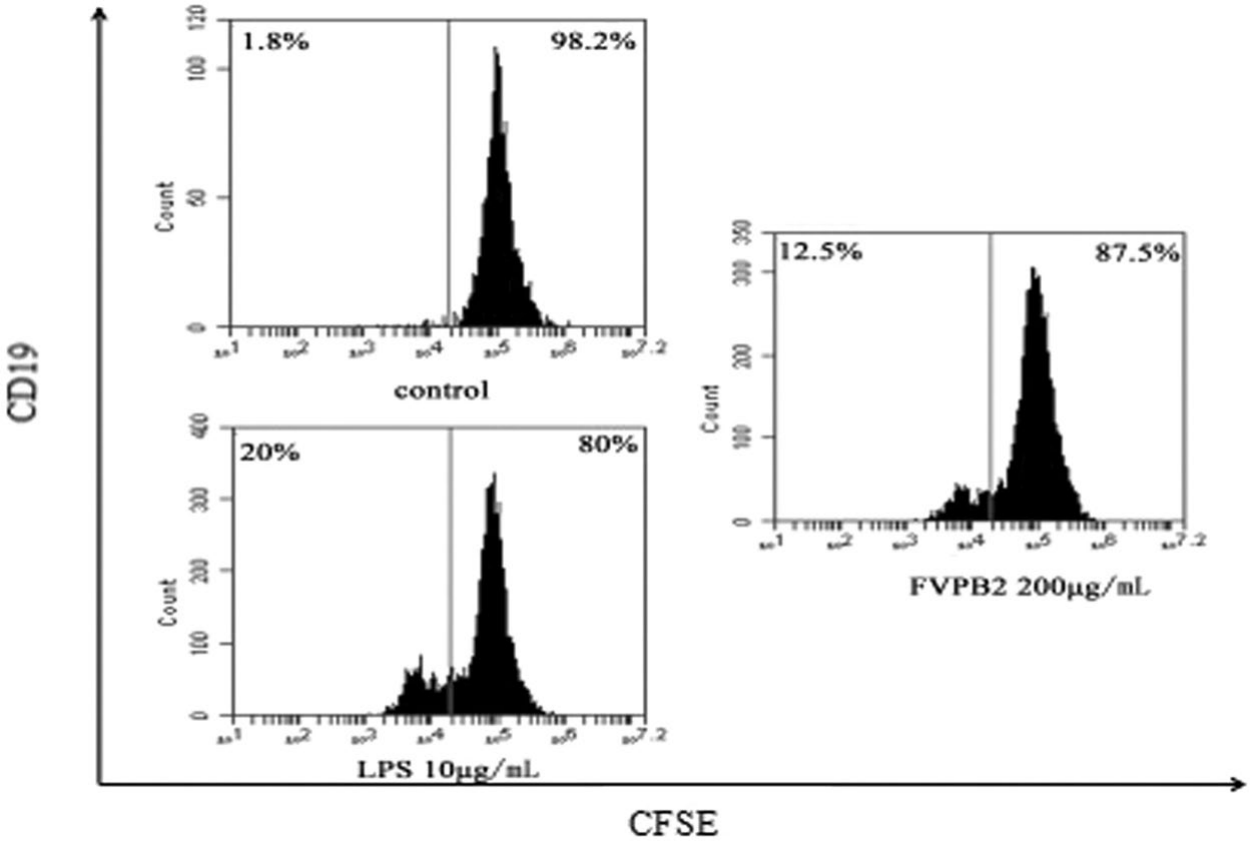
B cells isolated from mouse spleen-derived lymphocytes incubated with 200 μg/mL FVPB2 for 4 days. Proliferation ratio of B cells was detected by FACS. Control cells were treated with PBS.

To study the effect of *F*. *velutipes* polysaccharide on B cell proliferation, FVPB2 was used to treat the mouse spleen B cells directly, which were purified from mouse spleen-derived lymphocytes using MS Columns with anti-CD19 MicroBeads. The result of FACS showed that the percentage of B cells (CD19+/CD3−) was more than 90%. As shown in Fig. 5, the proliferation ratio of B cells treated with FVPB2 (200 μg/mL) increased more than 10.7% compared with the control group. Comparing Fig. 5 with Fig. 3B, these experiments proved that FVPB2 could promote B cells bioactivities.

### Effect of FVPB2 on activation of B cells isolated from mouse spleen lymphocytes

**Figure 6:**
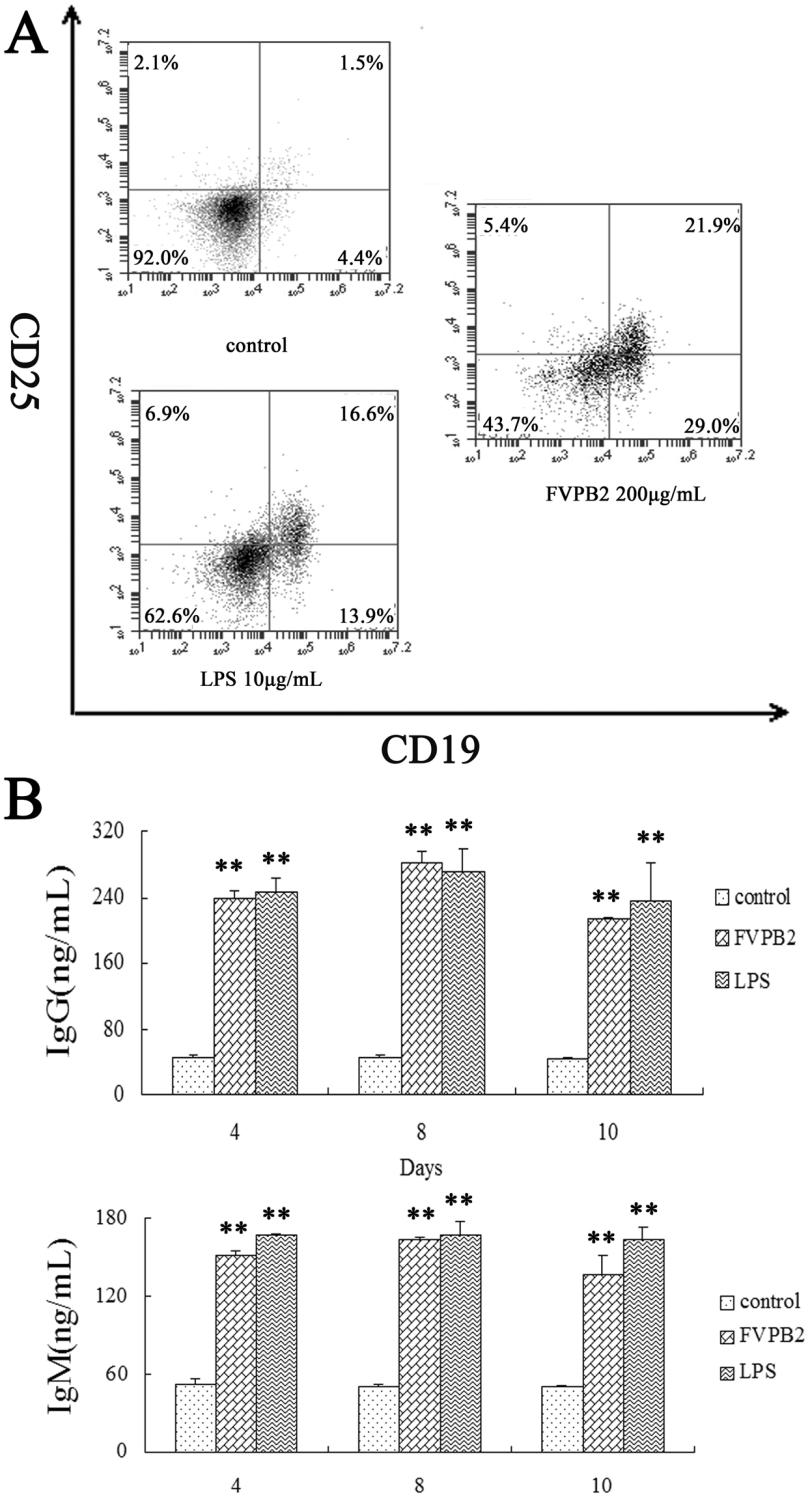
Activation of B cells isolated from mouse spleen lymphocytes treated by FVPB2. (**A**) Expression level of CD19 and CD25 on purified B cells, which were treated by 200 μg/mL FVPB2, 10 μg/mL LPS, or PBS for 48 h as analyzed by FACS. (**B**) Secretion of IgG and IgM by purified B cells treated with FVPB2. Purified B cells were incubated with 200 μg/mL FVPB2, 10 μg/mL LPS or PBS as control. Each value represents the mean ± SD of separate triplicate experiments. Values significantly higher than those of the controls are indicated by *P < 0.05 and **P < 0.01).

In order to identify the activation effect of FVPB2 on B cells, the expression of CD19 and CD25 on the surfaces of purified B cells were detected. The results revealed that the percentage of activated B cells (CD19^+^CD25^+^) increased significantly after treatment with 200 μg/mL FVPB2, which are similar to the experimental results in Fig. 6A.

The proliferation and activation effects of B cells were identified in the above experiments. The secretion of IgG and IgM by B cells was analyzed by ELISA. As shown in Fig. 6B, 200 μg/mL of FVPB2 enhanced the IgG and IgM release levels at 4, 8 and 10 days.

### Effect of IL-10 derived from regulatory B cells on B cells *in vitro*

**Figure 7:**
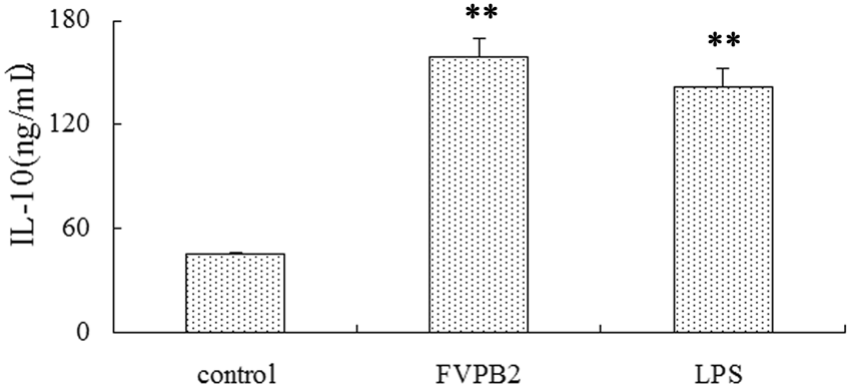
Effect of FVPB2 on IL-10 released by purified B cells was investigated by ELISA assay. The B cells were incubated with 200 μg/mL FVPB2, 10 μg/mL LPS or PBS (as control) for 4 days. Each bar represents the mean ± SD of three replications. Values with asterisks are significantly different (**P < 0.01 VS control).

To investigate the induction of FVPB2 on Interleukin 10 (IL-10) released by B cells, the expression level of IL-10 was determined by ELISA. Figure 7 shows that 200 μg/mL FVPB2 significantly enhanced the released level of IL-10 compared with control group.

The roles of extracellular regulated protein kinases (Erk1/2) and nuclear factor kappa-light-chain-enhancer of activated B cells (NF-κB) pathways on the IL-10 production by B lymphocytes cells were explored. IL-10 induction involves transcriptional activation via promoter binding of the transcription factors p38 and Activator protein 1 (AP-1)^23,24^. Purified B lymphocytes were chosen as a model to study the mechanisms. To assess whether FVPB2-induced IL-10 is dependent on ERK1/2 and NF-κB activation, purified B cells were preincubated with 5 μM ERK1/2 and 5 μM NF-κB inhibitor for 1 h respectively, followed by treatment with FVPB2 (200 μg/mL) for 3 days, and Brefeldin A, inhibitor of intracellular protein transport, was added for the last 5 h, and then the intracellular IL-10 was analyzed by FACS.

**Figure 8:**
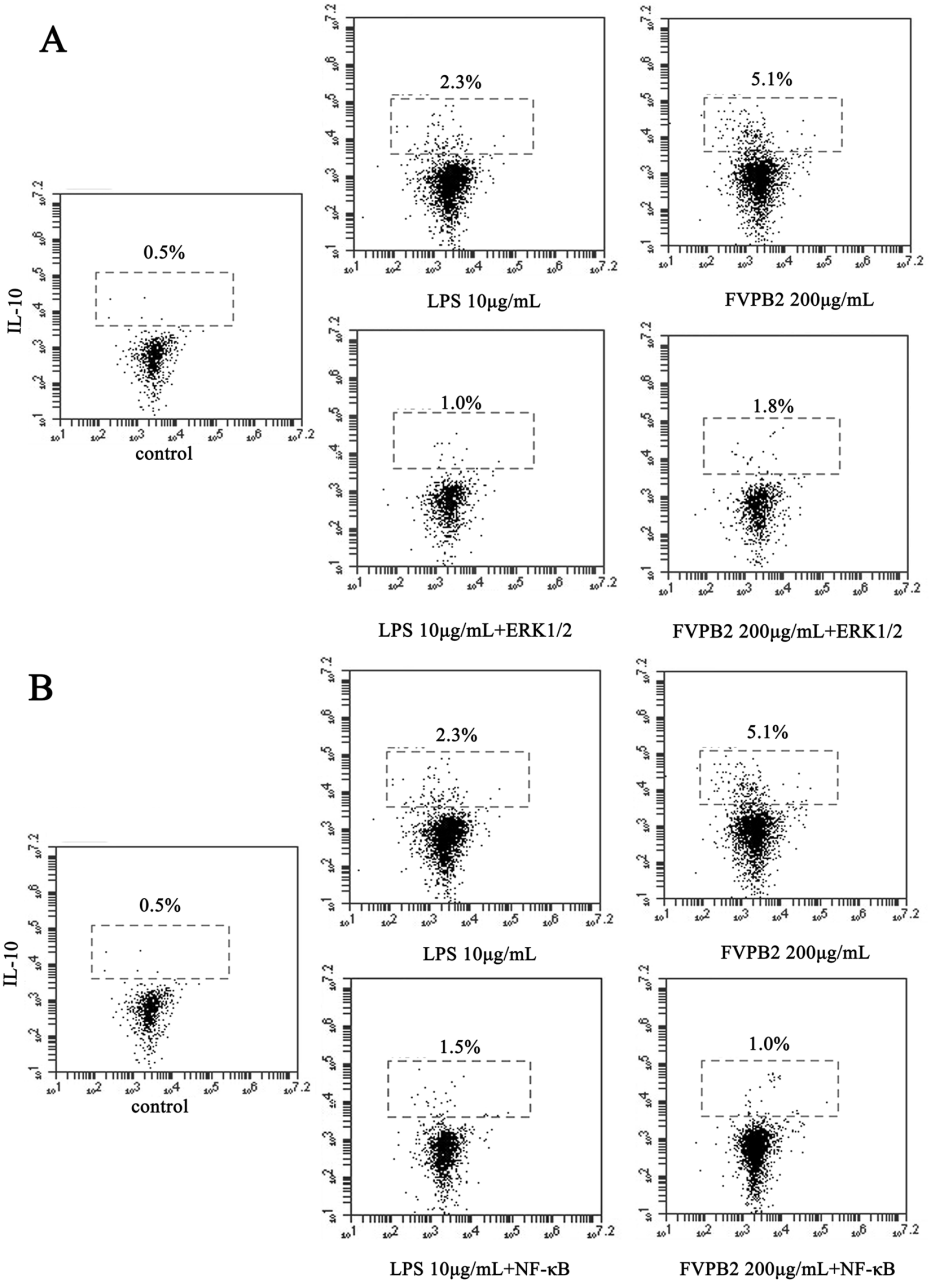
The influence of FVPB2 on the ERK1/2 and NF-κB signaling pathways. B cells were preincubated with 50 μg/mL ERK1/2 or 50 μg/mL NF-κB inhibitor for 1 h respectively, followed by treatment with 200 μg/mL FVPB2, 10 μg/mL LPS or PBS as the control for 3 days. Brefeldin A (BFA) was added for the last 5 h, and then the intracellular levels of IL-10 were analyzed by FACS.

ERK1/2 inhibitor attenuated IL-10 expression in B lymphocytes cells treated with 200 μg/mL FVPB2, and IL-10 production decreased from 5.1% to 1.8%, and the inhibitory ratio of IL-10 production was 64.71% (Fig. 8). As shown in Fig. 8, NF-κB inhibitor attenuated IL-10 expression in B lymphocty cells administrated with 200 μg/mL FVPB2, and IL-10 production decreased from 5.1% to 1.0%, and the inhibitory ratio of IL-10 production was 80.39%.

## Discussion

In this study, we have shown that FVPB2 with the molecular weights of ~1.50 × 10^4^ Da was separated using hot-water extract and isolated by DEAE-Sepharose Fast Flow (26 mm × 100 cm) and Sephacryl^TM^ S-300 gel chromatography (16 mm × 100 cm). Sugar analysis, methylation analysis, and 1D and 2D NMR spectroscopy revealed that FVPB2 was a new polysaccharide. Although several polysaccharide structures from *F*. *Veultipes* have been reported recently, the polysaccharide moiety of FVPB2 represents a previously undocumented novel structure^25–27^. Research is ongoing in our laboratory to further characterize the nature of the polysaccharide and to investigate structure-activity relationships.

We found the crude polysaccharide from *F*. *Veultipes* had anti-infamamtory effect, however, others reported the crude polysaccharides from *F*. *Veultipes* had pro-inflammatory effects. As it is known, the determination of the composition of crude polysaccharides are complicated, and many reports indicated that 3D-structure of a polysaccharide was closely related to biological activity^28,29^, consequently, the purified polysaccharide rather than crude polysaccharide should be used for biological activity research. Yan *et al*. reported a novel polysaccharide from *F*. *Veultipes* (MW ~ 140 kD) consisted of mannose, glucose, galactose, fucose and rhamnose in a molar ratio of 2:4:5:1:1, and could be bound to peritoneal macrophages and strongly stimulate them to produce NO *in vitro*^30^. Leung *et al*. reported polysaccharide (MW ~ 200 kD) was mainly composed of β-(1 → 3)-D-linked glucose, and could trigger proliferation of splenic lymphocytes^31^. Wang *et al*. reported two purified polysaccharides FVP-1 and FVP-2 (Crude polysaccharide (FVP) was isolated by hot water extraction, protein removal, pigments elimination and ethanol precipitation from *F*. *Veultipes*. FVP-1 and FVP-2 were obtained by DEAE ion-exchange chromatography and SephadexG-100 gel column chromatography from FVP.) were from *F*. *Veultipes* mycelium, which molecular weight were 1.48 × 10^4^ Da and 2.73 × 10^4^ Da, respectively^32^. FVP-1 and FVP-2 could stimulate proliferation of splenic lymphocytes *in vitro*^32^. Zhao *et al*. found that the combination of Zn and purified polysaccharide could change the immunoactivity of this polysaccharide, and exhibited its anti-inflammatory effect through inhibition of inflammatory cytokines release including IL-6, TNF-α, INF-γ and NO^33^. In this study, we demonstrated that FVPB2 had a pronounced stimulatory effect on the activation and immunoglobulin production of B cells. B cells could regulate immune system by secreting antibodies. And T2 B cells could present antigen (they are also classified as professional antigen-presenting cells (APCs)) and secrete cytokines. Mature B cells become plasma cells which secrete antibodies such as IgG and IgM. Removal of B cells from lung lymphocyte cultures resulted in diminished IFN-β secretion and tumor lysis, providing evidence for the importance of B cell responses in tumor defenses^34^. Ollert *et al*. discovered the presence of natural IgM antibodies in the sera of healthy adults. These antibodies elicit effective killing of neuroblastoma cells by both complement activation and apoptosis^35^. These results suggested that FVPB2 have anti-tumor activity mediated by the activation and immunoglobulin production of B cells.

Regulatory B cell (Breg) is a subpopulation of B cells that play immunomodulatory role in the immune system. This unique cell population has been found to inhibit other cells in the immune system that contribute to the development of autoimmune diseases and could prove promising in the treatment of autoimmune diseases^36^. In innate cells, these mechanisms include downregulation of proinflammatory cytokine production and decreased expression of MHC-II and co-stimulatory molecules resulting in decreased T cell activation^36^. Breg can contribute to the maintenance of tolerance via the expression of immune-regulatory cytokines such as IL-10^37^. The anti-inflammatory master regulator IL-10 is a multifunctional cytokine^38^. As shown in Fig. 7 and 8, purified B cells secreted IL-10 after being treated with FVPB2. However we haven’t a direct evidence for FVPB2 binding with the IL-10 receptor. As we known, IL-10 receptor 2 is a critical component of IL-10, and regulates IL-10-mediated immunomodulatory responses^39^. So whether IL-10 induction through the direct action of FVPB2 with the IL-10 receptor need to be explored in future. Some reports indicated IL-10 could regulate Ig production including IgG and IgM^40,41^. In our research, FVPB2 not only stimulated IL-10 release, but also increased IgG and IgM levels. Conceivably, these experimental results indicate FVPB2 regulated IgG and IgM expression through increasing the IL-10 level. Therefore, this new polysaccharide may possibly play an important role for the treatment of autoimmune and inflammatory diseases. To investigate the possible regulatory pathways in IL-10 expression, ERK1/2 and NF-κB inhibitors were added into the culture media of purified B cells, and IL-10 production was detected by FACS. ERK1/2 and NF-κB exist as inactive complex with class of inhibitory proteins. NF-κB, comprised of five members, ReIA (p65), Re1B, cRel, p50 and p52, is a major target and key player in inflammatory disease, which regulates various genes involved in immune and acute phase inflammatory responses^42–45^. Intracellular staining for interleukin-10 continues to be a consistent and reproducible method for identifying Breg in B cells. Figure 8 shows that ERK1/2 and NF-κB inhibitors influenced the expression of IL-10. The results clearly indicate that FVPB2 induction IL-10 is at least partially dependent on ERK1/2 and NF-κB activation in B cells.

In conclusion, our experimental results demonstrated that the new polysaccharide from *F*. *velutipes* (FVPB2) had typical immunostimulatory activity which was identified by promotional effects on the activation of B cells and release of IgG and IgM. In addition, our results indicate that FVPB2 had significantly upregulatory effect on IL-10 production, which has close relationship with Breg. It also indicates that this promotional effect on IL-10 production was regulated through the ERK1/2 and NF-κB signaling pathways. Accordingly, it is conceivable that the novel polysaccharide FVPB2 from *F*. *velutipes* has the potential to be an important new nutraceutical.

## Methods

### Materials and reagents

Fresh air-dried *F*. *velutipes* fruiting bodies were purchased from Shanghai Xuerong Bio-technology Co., Ltd (Shanghai, China). DEAE-Sepharose Fast Flow ion exchange and Sephacryl S-300 High Resolution media were purchased from GE Healthcare (Cardiff, UK). D-Gal, D-Ara, L-Fuc, L-Rha, D-Man, D-Xyl, D-Glc, D-glucuronic acid (GlcA), D-galacturonic acid (GalA), inositol and LPS (from *Escherichia coli* O55:B5 Sigma-Aldrich code: L2880-10MG) were all analytical reagent grade from Sigma-Aldrich (St. Louis, USA). RPMI-1640 medium and FBS was purchased from GIBCO (Grand Island, NY, USA). MS Separation Column and CD19 MicroBeads, mice were purchased from MASS Miltenyi Biotec (CA, USA). CellTrace^TM^ 5-(and 6)-Carboxyfluorescein diacetate succinimidyl ester (CFSE) Cell Proliferation Kit were purchased from Thermo Fisher (Waltham, MA). The Cytofix/cytoperm^TM^ Fixation/Permeabilization Kit and interleukin-10 (IL-10) were purchased from BD Biosciences (CA, USA). NF-κB inhibitor (SC75741) and extracellular signal-regulated kinase 1/2 (ERK1/2) inhibitor (PD98059) were purchased from Selleck Chemicals (Houston, USA). C57 mice were purchased from Model Animal Research Center of Nanjing University (Nanjing, China). IgG, IgM and IL-10 ELISA kits were purchased from Shanghai Jake Biological Technology Co., Ltd (Shanghai, China) AlamarBlue® (#BUF012B) was from AbD Serotec (Oxford, UK). All other reagents were analytical reagent grade from Sinopharm Chemical Reagent Co., Ltd (Shanghai, China).

### Extraction and purification of the FVPB2 polysaccharide

The fruiting bodies of *F*. *velutipes* were extracted with 95% ethanol for about 12 h to remove lipids. This step was repeated three times. After filtration, the residue was air-dried at room temperature, and then extracted with 100 °C distilled water twice (for 2 h each time). The liquid extracts were combined, centrifuged (26,000 × *g*, 20 min, 20 °C), concentrated to one-tenth of the original volume, and precipitated by adding 95% (v/v) ethanol until the final alcohol concentration reached 30%. The precipitated crude material was then washed with 95% (v/v) ethanol, resuspend in distilled water to the original volume, precipitated once more with 95% (v/v) ethanol, collected by centrifugation (26,000 × *g*, 20 min, 25 °C), and lyophilized, defined as FVP30. The FVP30 was washed with 95% (v/v) ethanol, resuspended in distilled water to its original volume, precipitated with 95% (v/v) ethanol again, and then collected by centrifugation (26,000 × *g*, 20 min, 25 °C), and then freeze dried. One gram of freeze dried material was resuspended in 100 mL of distilled water, and centrifuged as above. This solution was dialyzed (cut-off at 8.0–10 kDa) against running distilled water for 2 days, concentrated to 100 mL under reduced pressure at 40 °C and then centrifuged as before. The supernatant was applied to a DEAE-Sepharose Fast Flow column (XK26 × 100 cm), eluted first with distilled H_2_O and then with a 0–2 M gradient of NaCl. The fractions were collected by an auto-collector, and the carbohydrate fraction was detected by phenol–sulfuric acid method^46^. FVP30B was obtained from the 0–2 M NaCl gradient eluate. FVPB2 (0.5 g, yield 0.59%) was purified by gel-permeation chromatography on a column of Sephacryl S-300 High Resolution (XK16 × 100 cm) from FVP30B.

### Sugar analyses

Sample (2 mg) was hydrolyzed with 2 M trifluoroacetic acid (TFA) at 110 °C for 4 h according to Hardy *et al*.^47^. The monosaccharide composition was determined by high performance anion exchange chromatography (HPAEC) using a Dionex LC30 equipped with a CarboPac™ PA20 column (3 mm × 150 mm). The column was eluted with 2 mM NaOH (0.45 mL/min) followed by 0.05 to 0.2 M NaAc and the monosaccharides were monitored using a pulsed amperometric detector (Dionex). Monosaccharide components were determined using D-Gal, D-Glc, D-Ara, L-Fuc, L-Rha, D-Man, D-Xyl, D-GalA, and D-GlcA as standards.

### Determination of purity and molecular weight

FVPB2 was dissolved into phosphate buffer (0.15 M NaNO_3_ and 0.05 M NaH_2_PO_4_, pH 7) (2 mg/mL) to analyze the homogeneity and molecular weight by HPSEC. The system consisted of Waters 2695 HPLC system equipped with multiple detectors: refractive index detector (RI) and a UV detector for concentration determination, multiple angle laser light scattering detector (MALLS, DAWN HELEOS, Wyatt Technology, USA) for direct molecular weight determination and differential pressure viscometer (DP) for viscosity determination. The columns were TSK PWXL 6000 and 3000 gel filtration co lumns which were eluted with PB buffer at the flow rate of 0.5 mL/min. The column was calibrated using pullulan standards, P5 (6,200 Da), P10 (10,000 Da), P20 (21,700 Da), P100 (113,000 Da), P200 (200,000 Da) (Shodex, Japan). The column temperature and RI detector temperature were maintained at 35 °C.

### Infrared spectroscopy

Aliquots of FVPB2 (1 mg) were made into KBr discs and analyzed in a Perkin-Elmer 599B FT-IR spectrophotometer (USA).

### Methylation and GC-MS analyses

Methylation analysis of FVPB2 (2 mg) was conducted according to the method previously reported^48^. The methylated polysaccharide was then converted into partially methylated alditol acetates (PMAA) by hydrolysis, reduction with sodium borodeuteride (NaBD_4_), and acetylation, followed by linkage analysis using a GC-MS system (Thermo Finnigan TRACE 2000/MS) equipped with a DB-5 MS column (30 m × 0.25 mm, 0.25 µm, 0.2 mm film thickness, temperature programmed from 180 to 270 °C at 20 °C/min, and held at 270 °C for 25 min^49^. The individual peaks of the PMAA and fragmentation patterns were identified by their mass spectra and relative retention time in NIST 2011 database of GC-MS. The percentage of methylated sugars was estimated as ratios of the peak areas.

### NMR analysis

Prior to the measurements, samples were deuterium exchanged three times by freeze drying from D_2_O. ^1^HNMR (500 MHz, 27 °C) and ^13^C NMR (125 MHz, 27 °C) spectra were recorded with a Varian INOVA 500 NMR spectrometer (USA). Chemical shifts are referenced to the HDO resonance (δ4.78) at 27 °C as internal standard. The ^1^H-^1^H COSY, TOCSY, and (HMQC) were used to assign signals. HMBC and NOESY were used to assign inter-residue linkages and sequences.

### Preparation of lymphocytes from mouse spleens

All experiments involving animals and their care were conducted in conformity with NIH guidelines (NIH Pub. No. 85-23, revised 1996) and were approved by Animal Care and Use Committee of the Shanghai Academy of Agricultural Sciences, Shanghai China. All experiments with animal cell lines were performed in accordance with approved guidelines, and all experimental protocols were approved in accordance with the regulations established by the Shanghai Academy of Agricultural Sciences, Shanghai China.

C57 mice, 8–10 weeks of age (ca. 23 ± 1 g), were used for lymphocyte preparation. The animals were treated according to the Institutional Animal Care and Use Committee (IACUC) guidelines. The mice were killed by cervical dislocation. The spleens were subsequently removed and cut into several pieces, and then pressed through a stainless steel mesh (100 meshes) into a culture plate using a syringe plunger. The mesh was rinsed twice with PBS under sterile conditions. The spleen cell suspension was transferred to a new tube and precipitated for 10 min. The supernatant was pipetted into another tube and the cell clumps at the bottom of the tube were discarded. After centrifugation at 400 × *g* for 6 min, cell pellets were washed twice with PBS. In order to lyses red cells, cell pellets were resuspended for 10 min at room temperature in 1 mL Tris-HCl-buffered NH_4_C1 solution pH 7.2 [mix 9 volumes of 0.83% (w/v in water) NH_4_C1 with 1 volume of Tris-HCl (2.06% w/v in water, pH 7.65), adjust to pH 7.2]. Cells were counted in a Z series Counter (Counter Electronics, Miami, USA). The cell suspension was further diluted with a five-fold excess of medium. After mixing and centrifugation, the cell pellets were finally resuspended in RPMI 1640 medium containing 10% FBS **(**Grand Island, NY, USA).

### Determination of the proliferation of mouse lymphocytes by the AlamarBlue® Assay

Lymphocytes were adjusted to a concentration of 2 × 10^6^ cells/mL. After incubation at 37 °C in 5% CO_2_ atmosphere for 24 h, the medium was removed, and 200 µL RPMI 1640 medium containing 200 µg/mL FVPB2 were added into each well. RPMI 1640 containing 10 µg/mL LPS served as positive control. After incubation for 72 h, 20 μL AlamarBlue® was added to each well, and incubated for 6 h. When the medium color changed, the absorbance at 570 nm and 600 nm were measured using a spectrophotometric plate reader (Bio-Tek Instruments, Inc, Winooski, VT, USA). The proliferation rate was calculated according to the following formula:

$$\begin{array}{rcl}Proliferation\,rate & = & [117216\ast {A}_{570}-80586\ast {A}_{600}(sample)]\\  &  & /[117216\ast {A}_{570}-80586\ast {A}_{600}(control)]\end{array}$$

### Determination of mouse lymphocytes proliferation by CellTrace^TM^ CFSE Cell Proliferation Kit

CFSE fluorescent dyes were dissolved in DMSO as 5 mM stock solutions (stored at −20 °C). Lymphocytes were mixed with 1 μL of stock solution at a final concentration of 10^6^ cells/mL, and incubated in the dark for 20 minutes at room temperature. Cells were washed five times with RPMI 1640 (containing 10% FBS), and adjusted to a concentration of 2 × 10^6^ cells/mL. Fresh RPMI 1640 medium containing 200 µg/mL FVPB2 were added into each well of 96-well microplate. After incubation at 37 °C in a 5% CO_2_ atmosphere for 48 h (lymphocytes) or 96 h (B cells), the cells were analyzed by FACS.

### Analysis of the percentage of activated B cells in spleen lymphocytes using FACS

Lymphocyte cell suspension at a concentration of 2 × 10^6^ cells/mL (960 μL) and 40 μL of test agents were added to 24-well plates. Fresh RPMI 1640 medium containing 200 µg/mL FVPB2 were added into each well of 24-well microplate. After incubation for 48 h, lymphocytes were centrifuged, and washed twice with PBS (containing 1% BSA), and then labeled with different mAbs in PBS (containing 1% BSA) in the dark for 30 min at room temperature. After washing twice with PBS (containing 1% BSA), cells were resuspended in 0.3 mL of PBS, and sorted using BD AccuriC6 flow cytometery(BD FACSCalibur TM, Becton Dickinson, USA). FlowJo software **(**TreeStar, Ashland, OR) was used to analyze the percentage of various lymphocyte subpopulations. PE-conjugated anti-mouse CD19 conjugate, APC-conjugated anti-mouse CD25 and isotype control antibodies were from BD Biosciences (CA, USA).

### Separation of B cells from mouse spleen lymphocytes by magnetic cell sorting

Lymphocytes were prepared from mouse spleens, washed with PBS and supernatant removed completely, and 10^7^ cells were resuspended in 500 μL of buffer (PBS containing 0.5% BSA) and centrifuged at 300 × *g* for 10 minutes. After completely aspirating supernatant, cell pellets were suspended in 90 μL of buffer per 10^7^ cells, and 10 μL of magnetic cells sorting (MACS) CD19 Microbeads were added into 10^7^ cells. Cell suspension was mixed well and refrigerated at 4 °C for 15 min. Supernatant was removed completely, and cells were resuspended with buffer to a concentration of 2 × 10^8^ cells/mL. Then the cell suspension was transferred to LS + separation column which had been washed with 5 mL PBS and placed in the MidiMACS magnet. The cell suspension was run through the column, and the effluent was collected as non-B cells. Then the column was rinsed with 3 mL of buffer three times and then removed from the magnetic separator. Finally, 5 mL of buffer was added to the reservoir of LS column and the B cells were flushed out firmly using a plunger.

### Quantification measurement of IgG, IgM and IL-10 using ELISA Kits

The IgG and IgM released by the B cell lymphocytes were measured by ELISA. Lymphocytes were adjusted to a concentration of 2 × 10^6^ cells/mL. Fresh RPMI 1640 medium containing 200 µg/mL FVPB2 were added into each well of 96-well microplate. RPMI 1640 containing 10 µg/mL LPS served as positive control. Incubating at 37 °C in a 5% CO_2_ atmosphere for 4 days, 8 days and 10 days, supernatant of each sample was measured using IgG and IgM ELISA kits according to the manufacturer’s instructions.

The IL-10 production released by the B cells was measured by ELISA. B cells were adjusted to a concentration of 2 × 10^6^ cells/mL. To each well of a 96-well microplate 180 μL of cell suspension and 20 μL of test agent were added. After incubation at 37 °C in a 5% CO_2_ atmosphere for 4 days, supernatant was measured using IL-10 ELISA kit according to the manufacturer’s instructions.

### Effect of ERK1/2 (5 μM) and NF-κB (5 μM) inhibitors on IL-10 released from regulatory B cells detected by FACS

Intracellular IL-10 analysis by FACS was performed as described previously^50^. Purified mouse spleen B cells were adjusted to a concentration of 5 × 10^5^ cells/mL. Cells (180 μL) were preincubated with ERK1/2 inhibitor (5 μM) or NF-κB inhibitor (5 μM) for 1 h. Fresh RPMI 1640 medium containing 200 µg/mL FVPB2 were added into each well of 96-well microplate, and then incubated at 37 °C in a 5% CO_2_ atmosphere for 3 days. BFA was added for the last 5 h. Cells were blocked with anti-CD16/CD32 Fc-Block (Affymetrix eBioscience), fixed and permeabilized using the Cytofix/Cytoperm kit (BD Biosciences), and stained with anti-mouse PE-conjugated IL-10 (APC) mAb (BD Biosciences).

### Statistical analysis

All experiments were carried out in triplicate and data were presented as mean ± standard deviation (SD). Intergroup comparisons were performed by one-way analysis of variance (ANOVA) and LSD’s test. All of the variables were tested for normal and homogeneous variance by Levene’s test. When necessary, Tamhane’s T2 test was performed. A P value of less than 0.05 or 0.01 is significant and very significant, respectively.

### Informed Consent

All experiments involving animals and their care were conducted in conformity with NIH guidelines (NIH Pub. No. 85-23, revised 1996) and were approved by Animal Care and Use Committee of the Shanghai Academy of Agricultural Sciences, Shanghai China.
